# P01-030 – Proteinuria in FMF – prediction of nephropathy type

**DOI:** 10.1186/1546-0096-11-S1-A34

**Published:** 2013-11-08

**Authors:** OL Kukuy, A Livneh, A Ben-David, J Kopolovic, A Volkov, Y Shinar, E Holtzman, D Dinour, I Ben-Zvi

**Affiliations:** 1 Institute of Nephrology and Hypertension, Sheba Medical Center, Tel Hashomer, Ramat Gan, Israel; 2 Sackler Faculty of Medicine, Tel Aviv University, Tel Aviv, Israel; 3 Heller Institute of Medical Research, Sheba Medical Center, Tel Hashomer, Ramat Gan, Israel; 4 Department of Pathology, Hadassah-Hebrew University Medical Center, Jerusalem, Israel; 5 Department of Pathology, Sheba Medical Center, Tel Hashomer, Israel; 6 Heller Institute of Medical Research, Sheba Medical Center, Tel Hashomer, Ramat Gan, Israel

## Introduction

Reactive (AA) amyloidosis may complicate Familial Mediterranean fever (FMF), the prototype of autoinflammatory diseases. Thus, proteinuria in FMF is commonly viewed as resulting from amyloidosis and kidney biopsy is deemed superfluous. However, nephropathy other than amyloidosis has been described in FMF, but its rate and distinctive characteristics are unknown.

## Objectives

To determine the rate and underlying pathology of FMF related non-amyloidotic proteinuria and compare its clinical course, demographic and genetic features to those of FMF-amyloid nephropathy.

## Methods

This study is a retrospective analysis of data from all FMF patients, undergoing kidney biopsy for proteinuria above 0.5 gram/24 hrs, during 10 years (2001- 2011). Clinical, laboratory, genetic and pathology data were abstracted from patient files. Biopsies were viewed by an experienced pathologist, as necessary.

## Results

From 27 patients referred to kidney biopsy, only 16 (59.3%) were diagnosed with amyloid kidney disease (AKD), 11 were diagnosed with another nephropathy. The AKD and non amyloid kidney disease (NAKD) groups were comparable on most variables, but showed distinct characteristics with regard to the range of proteinuria (6.46 ±4.3 g vs. 2.4±1.7 g, p= 0.0136), rate of severe FMF (14 vs. 5 patients, p= 0.03) and rate of development of end stage renal disease (75% vs. 27.2%, p=0.02) respectively.

## Conclusion

NAKD is common in FMF and is featured with milder course and better prognosis. Contrary to common practice, it is highly suggested to obtain kidney biopsy from patients with FMF and proteinuria more than 0.5 gr/24 hrs.

## Disclosure of interest

None declared.

